# Understanding where parents take their sick children and why it matters: a multi-country analysis

**DOI:** 10.9745/GHSP-D-13-00023

**Published:** 2013-11-08

**Authors:** Stephen Hodgins, Thomas Pullum, Leanne Dougherty

**Affiliations:** a Save the Children, Washington, DC, USA; b ICF International, Calverton, MD, USA; c John Snow, Inc., Washington, DC, USA

## Abstract

**Background:**

Developing effective context-specific strategies to ensure that a high proportion of children receive timely and appropriate care requires knowing the source from which care is sought. Although Demographic and Health Surveys (DHS) and Multiple Indicator Cluster Surveys (MICS) collect such data in disaggregated form, they are not made available in the standard DHS and MICS reports.

**Methods:**

Secondary data analysis was done on 42 DHS and MICS surveys conducted since 2005 for care seeking for acute respiratory illness (DHS and MICS), diarrhea (DHS only), and fever (DHS only), disaggregating by urban-rural settings. Eight categories were used for source of care. Stata, version 12, was used for the analysis.

**Results:**

Patterns varied considerably, with care seeking in most of sub-Saharan Africa predominantly from public-sector providers, in South Asia predominantly from the private sector, and in Southeast Asia from a mix of public and private sources. Community health workers were not an important source of care.

**Conclusions:**

Variation in care-seeking patterns has implications for effective strategy, as described in more detail in 5 country examples from Asia and Africa. The analysis also suggests that it may be inappropriate to focus program efforts on community health workers to the exclusion of more widely used sources of care. The authors argue that, in order to ensure sounder program approaches, disaggregated care-seeking data should be routinely included in DHS and MICS reports. Finally, the authors call for more data on actual care provided in order to improve quality of care.

## Abstract (precis)

To effectively reach children with potentially life-threatening illness with needed treatment, it is important to understand where parents seek care. Data from 42 DHS and MICS surveys conducted since 2005 show that a majority of care in Africa is sought from the public sector; in South Asia, from the private sector; and in Southeast Asia, from a public-private mix. We recommend that such data be made available in standard DHS and MICS reports.

## BACKGROUND

In less-developed countries, pneumonia and diarrhea remain the leading causes of deaths among children beyond the newborn period; in many sub-Saharan African countries (particularly in West Africa), they are joined by malaria as a major cause.^1^ Preventive interventions are available for each of these conditions, but timely and appropriate *treatment* remains a fundamentally important program element. The key first steps in its successful delivery are recognition by the caregiver and care seeking outside the home.

In order to develop child survival strategies that effectively address the need for treatment of potentially life-threatening childhood illness, program managers need to understand the populations they work with. Indeed, it is to those developing and managing child health programs, particularly at the country level, that this paper is primarily directed. They need answers to key questions:

- To what extent are caregivers recognizing illness and seeking outside care?
- When they do seek outside care, where do they go?
- When care is sought, what actual treatments are received?

With such information, program managers are better enabled to develop strategies that respond to the challenges of their specific settings.

The Demographic and Health Surveys (DHS) and Multiple Indicator Cluster Surveys (MICS) have long included questions on child illness and care seeking. Questions are asked to elicit a recent history of cough, fever, and diarrhea. For each of these 3 categories of illness, the caregiver is then asked if advice or treatment was sought outside the home and, if so, from what source. In standard DHS reports, the one way this information is used is to generate an indicator for “care seeking from an appropriate provider.” The numerator for this indicator consists of cases for which advice or treatment was sought from a category of provider considered able to provide competent care (health facility or professional health worker). However, although the DHS (and MICS) data sets include details on specific sources of care, this is not presented in the standard reports. This is unfortunate because such information is needed to develop strategies that respond to the actual situation on the ground, with respect to current source of care.

Child health program managers need answers to key questions about care seeking in order to respond effectively to the challenges of their specific settings.

This paper reports on a secondary analysis of standard DHS surveys conducted in sub-Saharan Africa, South Asia, and Southeast Asia since 2005–2006 (excluding small-population island nations), and of MICS surveys conducted over the period 2005–2008, providing a disaggregated picture of care seeking for cough, fever, and diarrhea.

## METHODS

The data come from surveys conducted by Measure DHS, a project of the Bureau for Global Health at the U.S. Agency for International Development (USAID), and by the United Nations Children's Fund (UNICEF). All of the data sets are available online (at www.measuredhs.com and www.childinfo.org), along with country reports that include basic analyses of child health data, the questionnaires, and other documentation.

The authors analyzed care-seeking data on acute respiratory illness, diarrhea, and fever for 42 countries.

Information about treatment for possible acute respiratory infection (as this is operationalized in DHS and MICS studies) was reported for an average of 695 children in 24 DHS surveys, a total of 16,682 children, and in an additional 13 MICS surveys (with an average of 288 children for a total of 5,878 children). Fever was reported for an average of 2,295 children from 29 DHS surveys, a total of 66,549 cases. Information on diarrhea comes from the same 29 surveys, with an average of 1,369 children under 5 reporting diarrhea symptoms in the past 2 weeks in each of the surveys, giving a total of 39,393 cases.

Although advice or treatment-seeking questions were equivalent in the 2 survey types for acute respiratory infection (ARI), they were not for diarrhea and fever. MICS questions did not ask where the mothers sought advice or treatment, and questions focused instead on *sources for specific treatments*. For diarrhea, the MICS asked, “Where did you get the ORS [oral rehydration salts] packet?” For fever, the survey asked, “Where did you get the anti-malarials?” Because of this lack of comparability with the DHS questions, analysis on care seeking for diarrhea and fever was done using only DHS data.

The *structure* of the portion of the questionnaire on care seeking was essentially the same in all surveys (for ARI, in both DHS and MICS, and for diarrhea and fever in DHS). The options for *place of treatment* were similar but not identical: in each of the surveys, specific types of health facility or provider, both formal and informal, thought to provide at least some sick-child care in that country setting were included as possible response categories. Because these categories varied across surveys, for our purposes it was necessary to form a set of general categories to which these responses could be mapped:

- Public-sector hospital
- Public-sector peripheral health facility (non-hospital), including mobile/outreach clinics
- Private-sector health professionals, clinics, and hospitals
- Community health workers
- Services provided by faith-based organizations and other NGOs
- Retail outlets (only), including pharmacies, patent medicine shops, vendors
- Non-allopathic providers

We also included an “other” category for responses that did not fit in any of the above, and we created a new variable for “any public provider,” which included cases for which care was sought from one of the first 2 categories above. Note that with the exception of retail outlets, the categories used were *not* mutually exclusive so if, for the same case, advice or treatment was sought from providers from more than 1 category, that case would contribute to both values. Retail outlets were treated differently because we wanted to focus on episodes for which advice or treatment was sought from such outlets alone and so did not include those in which the shop simply filled a prescription based on advice received from another category of provider.

Because the survey question is administered in a way that can elicit more than 1 source of care per episode, if, in fact, more than 1 was consulted, summing the proportions across all sources in most cases yields a slightly larger number than the total proportion of cases for which care was sought.

Although the interviewers were instructed to probe and include multiple sources, it is likely that there was some variation from one interviewer to another, as well as from one survey to another, in the extent to which multiple sources were identified.

Note that all n's and percentages in the tables are weighted. DHS and MICS surveys use a stratified cluster design, typically with strata consisting of all combinations of region (the first subnational unit) and place of residence (urban/ rural). The weighting procedures are built into the software package used. Programming was done primarily with version 12 of Stata.

Supplementing the analysis disaggregating by type of provider, we have further explored aspects of care rendered by different categories of provider for insights into quality or appropriateness. For ARI, in DHS surveys that asked both about source of care and whether antibiotics were dispensed, we determined the proportion of cases receiving antibiotics, disaggregating by “appropriate” or “medically trained” providers (government health facilities, NGO health facilities, private physicians or health facilities, community health workers) vs. “non-appropriate” providers (drugs shops or pharmacies only and non-allopathic providers). In DHS, the ARI category is used as a reasonable proxy for possible pneumonia, potentially warranting antibiotic treatment. As such, the proportion of such cases for which antibiotics are given is a commonly used population indicator for adequacy of reach of pneumonia treatment services. Certainly, some proportion of such cases would not have had symptoms or signs at the time of examination that should prompt a competent clinician to prescribe antibiotics; however, very low rates of antibiotic treatment among cases seen by a health worker would suggest a systemic problem with adequacy of care.

For diarrhea care, we looked at 2 measures of quality. First, we determined the proportion receiving ORS, disaggregating by “appropriate” vs. “non-appropriate” provider. Second, restricting to cases of reported non-bloody diarrhea, we determined the proportion reporting using pills or syrups (excluding zinc). In most settings, these would include antibiotics and anti-motility agents, although, of course, this category could include many other remedies. We did not include cases of bloody diarrhea, for which antibiotic treatment would be warranted (and a response indicating use of pills or syrups could represent appropriate care). For the retained non-bloody diarrhea cases, current treatment recommended by the World Health Organization (WHO) includes only zinc and ORS. Therefore, we interpret the dispensing of other products for non-bloody diarrhea as representing a sub-optimal practice.

## RESULTS

### Overview

Detailed results for source of care for ARI, fever, and diarrhea are presented in Table 1, Table 2, and Table 3, and for quality of ARI and diarrhea care in Table 4 and Table 5. The map (see Figure) provides a clearer spatial picture of similarities and differences across countries and regions. As is evident from the map, a pattern of reliance primarily on public-sector sources is predominant across Africa, with several notable exceptions (Nigeria, Somalia, and Uganda). In South Asia, by contrast, the private sector is the major source. In Southeast Asia there is a more mixed pattern for sources of care. In general, care-seeking levels were high in the Asian countries (with the exception of the Philippines and the 2 Central Asian Republics included in the analysis), and mortality was lower than in the African countries.

**Figure.**
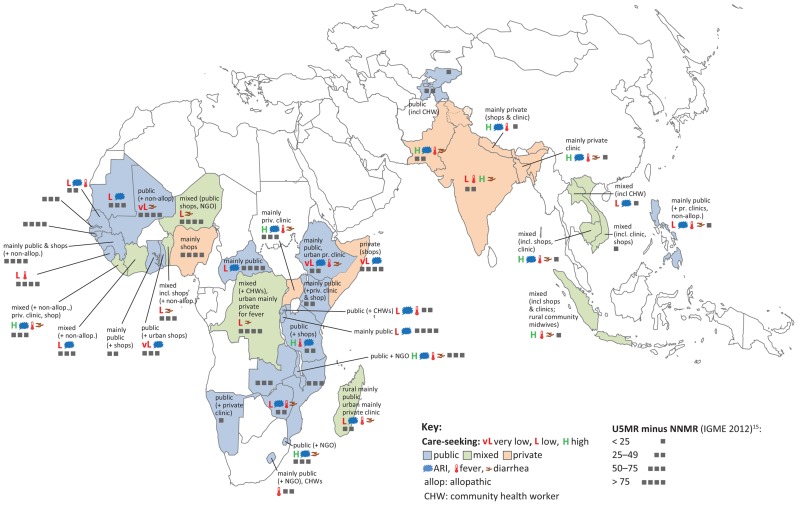
Sources of Care Seeking for Childhood Illness 1. 42 countries are included in the map. 2. For purposes of characterizing the pattern of care seeking, public hospital, public outpatient health facility, and community health worker (CHW) are grouped to form “public.” Similarly, “private clinic” (which includes private hospital, private clinic, and private clinician) is grouped with retail sources, referred to here as “shop,” to form the category “private.” 3. Comparing the proportion seeking care between two categories, if the difference is greater than 40 percentage points, the care-seeking pattern is characterized as simply “public” or “private” (more specifically “private clinic” or “shop” if that specific source is predominant). 4. If the difference is from 10–40 percentage points, the adjective “mainly” is used. 5. If the difference is less than 10, the term “mixed” is used. 6. Additional sources of care are mentioned if a percent threshold is met, notably: at least 5% for non-allopathic, CHW, or NGO; and at least 10% for “shop” or “private clinic.” 7. For the following countries, MICS data were used and covered only acute respiratory infection (ARI), not diarrhea or fever: Burundi, Central African Republic, Côte d'Ivoire, Guinea-Bissau, Kyrgyzstan, Laos, Mauritania, Mozambique, Somalia, Tajikistan, Togo, and Vietnam.

**Table 1.** Sources of Care for Acute Respiratory Infection (%)

		**Public Hospital**	**Public Periph HF**	**Private Clinic(ian)**	**CHW**	**NGO/ Religious**	**Pharmacy/ Shop Only**	**Non-Allopathic**	**Other**	**Any Public (not incl. CHWs)**	**Any Source**	**N^a^**
**Survey – DHS Africa**												

***West and Central Africa***												

Benin 2006	Urban	(4)	19	11		(4)	19	12	10	22	73	**453**
	Rural	(2)	24	6	(1)	3	14	12	10	26	66	**926**
	All	3	22	7	(1)	3	16	12	10	25	69	**1,379**
												
DR Congo 2007	Urban	(8)	16	18	(4)		23	(2)	(1)	24	65	**226**
	Rural	(4)	24	5	10		12	6	(2)	26	56	**336**
	All	5	21	10	7		16	4	(2)	26	60	**562**
												
Ghana 2008	Urban	(36)	(3)	(15)			(10)		(3)	(38)	63	**53**
	Rural	(14)	32	(4)			(15)	(1)	(2)	45	66	**97**
	All	22	22	(8)			(13)	(1)	(2)	43	65	**150**
												
Guinea 2005	Urban	(13)	40	(7)			(6)	(2)	(6)	51	72	**95**
	Rural	(3)	28	(2)	(5)		17	14	(1)	31	65	**356**
	All	(5)	30	(3)	(4)		15	12	(2)	35	67	**451**
												
Liberia 2007	Urban	(15)	43	(16)			(10)		(1)	58	83	**104**
	Rural	(8)	34	18			15	(4)	(4)	42	78	**336**
	All	10	36	18			14	(3)	(3)	46	80	**440**
												
Mali 2006	Urban	(5)	39	(6)	(1)		(12)	(6)	(4)	43	69	**188**
	Rural	(1)	32	(1)			(2)	11	10	32	56	**518**
	All	(2)	33	(2)	(1)		5	10	8	35	59	**706**
												
Niger 2006	Urban	(5)	42	(7)		(10)	(16)	(3)	4	46	79	**173**
	Rural	(1)	24			21	25	4		25	72	**1,034**
	All	(2)	26	(1)		19	23	4	(1)	28	73	**1,207**
												
Nigeria 2008	Urban	18	(6)	(9)			42	(3)		24	77	**172**
	Rural	9	13	8	(2)		36	(2)		22	66	**519**
	All	11	12	9	(1)		37	(2)		22	69	**690**
												
Senegal 2010–2011	Urban	14	37	9	(2)		(8)	(1)		50	69	**310**
	Rural	(3)	32	(1)	(2)		(4)	(6)	(4)	35	50	**278**
	All	9	35	(5)	(2)		(6)	(3)	(2)	43	60	**589**
												
Sierra Leone 2008	Urban	(8)	(29)	(11)	(2)		(9)			(36)	58	**56**
	Rural	(6)	33	(7)	(2)		(4)	(1)	(1)	38	51	**281**
	All	(6)	32	(7)	(2)		(5)	(1)	(1)	38	52	**337**
												
***Southern Africa***												
												
Lesotho 2009	Urban	(24)	(9)	(27)		(22)		(10)		(32)	(71)	**22**
	Rural	(8)	32	(7)	(4)	16	(5)	(2)		39	72	**162**
	All	(10)	29	(9)	(4)	17	(5)	(3)		39	72	**184**
												
Madagascar 2008–2009	Urban	(8)	(18)	(35)					(1)	(25)	61	**52**
	Rural		30	10			(7)	(2)	(1)	30	47	**292**
	All	(1)	28	14			(6)	(2)	(1)	29	49	**345**
												
Malawi 2010	Urban	34	21	(11)		(3)				54	68	**168**
	Rural	14	42	8		11	6	(1)	(1)	55	78	**1,053**
	All	17	39	9		10	6	(1)	(1)	55	76	**1,221**
												
Namibia 2006–2007	Urban	(29)	(34)	(22)			(4)		(1)	63	87	**52**
	Rural	22	37	(9)				(1)		58	67	**149**
	All	24	36	13			(1)	(1)		59	72	**201**
												
Swaziland 2006–2007	Urban	12	31	(6)	(3)	34	(4)			42	82	**32**
	Rural	(3)	(50)	(5)	(1)	(14)	(6)			(53)	(77)	**182**
	All	(5)	47	(5)	(2)	17	(6)			52	78	**214**
												
Zambia 2007	Urban	(10)	57	(1)			(10)			66	72	**95**
	Rural	(5)	54	(1)	(1)	(8)	(5)	(3)	(1)	59	75	**209**
	All	(7)	55	(1)	(1)	(6)	(6)	(3)	(1)	61	74	**304**
												
Zimbabwe 2010–2011	Urban	(3)	34	(6)			16			38	60	**38**
	Rural	(1)	43			(5)	(4)		(2)	(44)	(54)	**179**
	All	(2)	42	(1)		(4)	(6)		(1)	43	55	**217**
												
***East Africa***												
												
Ethiopia 2011	Urban	(6)	(15)	(28)		(2)	(3)			(19)	50	**69**
	Rural		19	5			2			20	27	**703**
	All	(1)	19	7			2			20	29	**773**
												
Kenya 2008–2009	Urban	(20)	30	(12)		(10)	(14)			47	79	**71**
	Rural	12	33	9		(2)	14	(1)	(1)	44	69	**346**
	All	13	33	10		(3)	14	(1)	(1)	45	70	**416**
												
Rwanda 2010	Urban		(45)	(21)	(10)		(6)		(2)	(45)	84	**54**
	Rural		35		14		(3)	(1)	9	35	58	**269**
	All		36	(4)	13		(4)	(1)	8	36	62	**322**
												
Tanzania 2010	Urban	(25)	47				(10)		(14)	70	85	**85**
	Rural	(6)	51				18		(5)	57	78	**247**
	All	11	50				16		8	60	80	**332**
												
Uganda 2011	Urban	(13)	(16)	55	(3)		(7)		(3)	28	90	**141**
	Rural	4	29	48	(2)	(1)	3	(1)	(1)	32	82	**977**
	All	5	27	49	2	(1)	4	(1)	(1)	32	83	**1,118**
												
**Survey – DHS Asia**												
												
Bangladesh 2011	Urban	(5)	(7)	57	(1)	(1)	(21)	(3)	(1)	(12)	91	**40**
	Rural	(3)	(9)	45	(1)		22	(6)	(1)	12	80	**237**
	All	(4)	(8)	47	(1)	(1)	22	(5)	(1)	12	82	**277**
												
Cambodia 2010	Urban	(17)	(24)	(26)			(22)		(9)	(39)	89	**41**
	Rural	(5)	21	20			18		24	26	84	**457**
	All	6	21	21			18		23^b^	27	85	**498**
												
India 2005–2006	Urban	12	4	60	(1)		3	3	1	16	81	**1,110**
	Rural	6	10	46	1		4	4	2	15	70	**4,762**
	All	7	9	49	1		4	4	2	15	72	**5,872**
												
Indonesia 2007	Urban	(3)	22	29	5	(1)	23		19	25	94	**686**
	Rural	(2)	23	11	16	(2)	24	(2)	19	25	91	**1,103**
	All	3	22	18	12	(1)	24	2	19	25	92	**1,789**
												
Nepal 2011	Urban	(28)	(5)	(43)	(1)		(26)			(33)	(89)	**24**
	Rural	(2)	20	19	(3)		31	(2)	(3)	22	77	**215**
	All	(5)	19	23	(2)		30	(2)	(2)	23	78	**238**
												
Pakistan 2006–2007	Urban	8	(1)	71	(1)		(1)	(3)	(6)	9	89	**323**
	Rural	8	(2)	56			(3)	(2)	12	10	81	**854**
	All	8	2	60			(2)	3	10	10	83	**1,178**
												
Philippines 2008	Urban	(13)	28	(18)			(2)	(5)	(1)	39	61	**127**
	Rural	(5)	30	13			(4)	15	(1)	35	60	**197**
	All	8	29	15			(3)	11	(1)	36	60	**324**
												
**Survey - MICS**												
												
Burundi 2006	Urban	(17)	(14)	(15)	(2)		(6)	(1)	(2)	31	(52)	**25**
	Rural	6	28	4			(1)	(1)	3	33	43	**1,061**
	All	6	27	4			(1)	(1)	3	33	43	**1,086**
												
Central Afr Rep 2006	Urban	19	12	(7)			(4)	(1)	11	29	48	**283**
	Rural	(4)	17	(5)	(4)		(3)	(3)	(4)	20	36	**389**
	All	10	15	6	(2)		(3)	(2)	7^c^	24	41	**672**
												
Côte d'Ivoire 2006	Urban	24	(19)	(13)	(2)		(5)	(12)	(7)	43	71	**121**
	Rural	(6)	16	(2)	(3)		(6)	13	17	22	57	**301**
	All	11	17	(5)	(3)		6	13	14^c^	28	61	**422**
												
Gambia 2006	Urban	(12)	43	(11)			(19)		(2)	54	79	**127**
	Rural	(8)	56	(7)	(4)		(7)	(2)	(2)	64	79	**236**
	All	10	51	8	(2)		11	(1)	(2)	60	79	**362**
												
Guinea Bissau 2006	Urban	45	26	(11)	(3)		(1)	(1)	(9)	63	76	**101**
	Rural	17	27	(2)	(5)		(1)	(1)	(8)	41	53	**148**
	All	28	27	(5)	(4)		(1)	(1)	(8)	49	62	**249**
												
Kyrgyzstan 2006	Urban	42	(27)		(1)		(6)			69	70	**73**
	Rural	(17)	48	(8)	(1)		(1)			57	58	**99**
	All	28	39	(4)	(1)		(3)			62	63	**172**
												
Laos 2006	Urban	(32)	(8)	(18)			(15)			(40)	(73)	**18**
	Rural	(9)	(13)	(11)	(9)		(6)		(3)	20	47	**172**
	All	(11)	(13)	(12)	(9)		(7)		(3)	22	50	**190**
												
Mauritania 2007	Urban	16	23	(8)	0		(9)			38	53	**264**
	Rural	9	22	(3)	(3)		(2)			31	36	**288**
	All	12	22	5	(2)		5			34	45	**552**
												
Mozambique 2008	Urban	(9)	59	(6)			(2)		(5)	66	77	**178**
	Rural		63	(4)	(1)				(5)	63	73	**356**
	All	(3)	62	(4)			(1)		5	64	74	**534**
												
Somalia 2006	Urban	(4)	10	11			28	(1)		13	51	**280**
	Rural	(1)	(2)	(4)	(2)		14	(1)	(1)	(3)	22	**558**
	All	(2)	4	6	(1)		18	(1)	(1)	6	32	**838**
												
Tajikistan 2005	Urban	(30)	(28)							(58)	(58)	**21**
	Rural	(23)	(41)		(13)					(57)	67	**43**
	All	(25)	(37)		(9)					57	64	**64**
												
Togo 2006	Urban	(5)	(12)	(6)			(1)	(1)	(7)	(18)	30	**34**
	Rural	(2)	15	(2)	(2)		(1)	(7)	(2)	17	30	**236**
	All	(3)	14	(3)	(1)		(1)	(5)	(4)	17	30	**270**
												
Vietnam 2006	Urban	36	(13)	45	(6)		(15)			49	99	**101**
	Rural	18	35	26	(6)		8	(1)	(3)	50	89	**366**
	All	21	31	29	6		9	(1)	(2)	50	90	**467**

a N values are adjusted (see Methods section). Note that point estimates for proportions based on adjusted Ns of less than 25 are indicated in parentheses, indicating a lower level of precision.

b This consisted mostly of “home of trained health worker.”

c In most of these cases, the reported source was “parent or friend.”

Abbreviations: CHW, community health worker; HF, health facility; NGO, nongovernmental organization.

**Table 2.** Sources of Care for Fever (%)

		**Public Hospital**	**Public Periph HF**	**Private Clinic(ian)**	**CHW**	**NGO/ Religious**	**Pharmacy/ Shop Only**	**Non-Allopathic**	**Other**	**Any Public (not incl. CHWs)**	**Any Source**	**N^a^**
**Survey – DHS Africa**												
												
***West and Central Africa***												
												
Benin 2006	Urban	(6)	20	12		4	17	9	10	25	73	**1,307**
	Rural	2	24	6	(1)	3	12	10	9	26	63	**2,854**
	All	3	23	8	(1)	3	14	10	10	26	66	**4,162**
												
DR Congo 2007	Urban	6	14	21	(4)		18	(1)	(4)	19	63	**950**
	Rural	(3)	21	6	10		10	4	(2)	24	54	**1,519**
	All	4	18	12	8		13	3	3	22	57	**2,469**
												
Ghana 2008	Urban	34	(8)	18			23			42	82	**197**
	Rural	16	25	(4)			15		(4)	41	64	**347**
	All	23	19	9			18		(3)	41	70	**544**
												
Guinea 2005	Urban	14	32	(7)	(2)		17	(4)	(4)	43	73	**376**
	Rural	(1)	24	(2)	(4)		19	11	(1)	26	59	**1,526**
	All	4	26	3	4		18	10	(2)	29	62	**1,902**
												
Liberia 2007	Urban	17	30	23			17	(2)	(2)	46	81	**450**
	Rural	6	26	21			15	9	7	32	74	**1,127**
	All	9	27	22			15	7	6	36	76	**1,577**
												
Mali 2006	Urban	(4)	37	(4)	(1)		12	12	(4)	41	72	**558**
	Rural		25		2		4	12	11	26	53	**1,680**
	All	2	28	(1)	2		6	12	9	29	57	**2,238**
												
Niger 2006	Urban	(6)	43	(7)		(9)	13	(1)	(3)	49	78	**324**
	Rural		25			18	18	3		25	62	**2,019**
	All	(1)	27	(1)		16	17	2	(1)	28	64	**2,343**
												
Nigeria 2008	Urban	19	10	12	(1)		33		(1)	28	74	**987**
	Rural	9	16	8	(1)		34	(1)	(1)	25	68	**2,981**
	All	12	14	9	1		34	1	1	26	70	**3,968**
												
Senegal 2010–2011	Urban	12	30	7	(2)		9	(1)	4	42	62	**1,211**
	Rural	3	31	(1)	2		5	3	2	34	46	**1,252**
	All	8	31	4	2		7	2	3	38	54	**2,463**
												
Sierra Leone 2008	Urban	(11)	17	18	(3)		(16)		(5)	28	69	**352**
	Rural	(4)	30	8	(2)		6	(1)		33	49	**931**
	All	6	26	11	(2)		9	(1)	(2)	32	55	**1,283**
												
***Southern Africa***												
												
Lesotho 2009	Urban	31	(11)	(8)		(7)	(5)	(2)		43	60	**112**
	Rural	(6)	26	9	(6)	16	(4)	(2)	(1)	32	68	**465**
	All	11	23	9	(5)	14	(4)	(2)		34	66	**577**
												
Madagascar 2008–2009	Urban	(5)	18	39			(2)			24	63	**164**
	Rural	(1)	29	8			5	(2)	(1)	30	45	**952**
	All	(2)	27	13			4	(1)	(1)	29	47	**1,116**
												
Malawi 2010	Urban	30	24	13		(5)	(3)		(1)	53	72	**786**
	Rural	11	40	5		10	8	(1)	(1)	50	73	**5,428**
	All	13	38	6		9	8		(1)	51	73	**6,214**
												
Namibia 2006–2007	Urban	15	25	17			(5)	(1)		40	63	**320**
	Rural	15	36	(5)			(2)	(1)		50	57	**476**
	All	15	32	10			(3)	(1)		46	60	**795**
												
Swaziland 2006–2007	Urban	(7)	(30)	(9)	(2)	(12)	(6)			36	61	**98**
	Rural	(3)	42	(4)	(1)	10	(5)		(1)	45	63	**605**
	All	(4)	40	(4)	(1)	10	(5)		(1)	44	63	**703**
												
Zambia 2007	Urban	9	54	(2)			(7)			63	71	**276**
	Rural	(3)	50		(2)	7	7	(2)	(2)	53	71	**768**
	All	5	51	(1)	(2)	5	7	(1)	(1)	55	71	**1,044**
												
Zimbabwe 2010–2011	Urban	(1)	25	(3)		(1)	12		(3)	25	44	**134**
	Rural	(1)	33	(1)		(4)	(4)		(1)	34	44	**372**
	All	(1)	31	(2)		(3)	6		(1)	32	44	**506**
												
***East Africa***												
												
Ethiopia 2011	Urban	(4)	19	16		(1)	(4)			22	41	**226**
	Rural	(1)	16	6		(1)	2	(1)		16	24	**1,659**
	All	(1)	16	7		(1)	2			17	26	**1,885**
												
Kenya 2008–2009	Urban	24	18	(9)		(4)	11			41	63	**223**
	Rural	10	29	8		(2)	14			39	62	**1,079**
	All	12	27	8		(2)	14			39	62	**1,302**
												
Rwanda 2010	Urban	(1)	29	(13)	(14)		(8)		(1)	31	65	**172**
	Rural		26		16		3	(1)	4	26	49	**1,183**
	All		26	2	16		3	(1)	4	27	51	**1,355**
												
Tanzania 2010	Urban	19	32	(2)			15		16	50	80	**454**
	Rural	(2)	52				23		4	53	78	**1,300**
	All	7	47				21		7	53	79	**1,754**
												
Uganda 2011	Urban	21	(11)	45	(3)		(4)			32	85	**330**
	Rural	5	24	45	5		4		(1)	28	83	**2,712**
	All	6	23	45	5		4		(1)	28	83	**3,042**
												
**Survey – DHS Asia**												
												
Bangladesh 2011	Urban	(3)	(8)	45		(1)	20	(3)	(1)	(11)	76	**70**
	Rural	(2)	(7)	43			21	(5)	(1)	8	74	**318**
	All	(2)	7	43			21	(4)	(1)	9	75	**388**
												
Cambodia 2010	Urban	(13)	(13)	26			30		(11)	25	86	**318**
	Rural	5	27	17			19		21	31	82	**1,877**
	All	6	25	18			20		19^b^	30	82	**2,194**
												
India 2005–2006	Urban	9	2	46			2	2		11	60	**1,954**
	Rural	4	7	36	(1)		4	2	2	11	52	**6,931**
	All	5	6	38	(1)		3	2	2	11	54	**8,885**
												
Indonesia 2007	Urban	(3)	25	25	6	(1)	21		19	27	93	**1,937**
	Rural	2	24	11	15	(1)	23	2	19	25	90	**3,096**
	All	2	24	16	12	(1)	23	2	19^c^	26	91	**5,033**
												
Nepal 2011	Urban	(13)	(6)	(36)	(1)	(1)	(27)		(1)	(19)	81	**91**
	Rural	(2)	15	22	(2)		30		(1)	17	71	**869**
	All	(3)	14	23	(2)		30		(1)	17	72	**960**
												
Pakistan 2006–2007	Urban	8	(1)	67	(1)		(2)	(4)	(4)	8	83	**791**
	Rural	9	(2)	51			(4)	(2)	14	11	80	**1,777**
	All	8	(2)	56			3	3	11	10	81	**2,569**
												
Philippines 2008	Urban	(6)	24	18			(2)	(6)		30	53	**586**
	Rural	(3)	22	10			(4)	10		25	46	**799**
	All	5	23	13			(3)	8		27	49	**1,385**

a N values are adjusted (see Methods section). Note that point estimates for proportions based on adjusted Ns of less than 25 are indicated in parentheses, indicating a lower level of precision.

b This consisted mostly of home of trained health worker.

c This consisted mostly of community nurse-midwives.

Abbreviations: CHW, community health worker; HF, health facility; NGO, nongovernmental organization.

**Table 3.** Sources of Care for Diarrhea (%)

		**Public Hospital**	**Public Periph HF**	**Private Clinic(ian)**	**CHW**	**NGO/ Religious**	**Pharmacy/ Shop Only**	**Non-Allopathic**	**Other**	**Any Public (not incl. CHWs)**	**Any Source**	**N^a^**
												
**Survey – DHS Africa**												
												
***West and Central Africa***												
												
Benin 2006	Urban	2	12	6		2	12	7		15	39	**393**
	Rural	1	15	2		2	11	10		16	41	**925**
	All	2	14	3		2	11	9		16	40	**1,317**
												
DR Congo 2007	Urban	(2)	22	(1)	8		4		(2)	24	34	**239**
	Rural	(1)	22		14		5		2	22	38	**360**
	All	(1)	22		12		4		2	23	37	**599**
												
Ghana 2008	Urban	22	(8)	(8)			28		(4)	29	66	**181**
	Rural	17	22	(4)			19		(2)	39	62	**361**
	All	18	17	5			22		(2)	36	63	**542**
												
Guinea 2005	Urban	8	31	(6)	(1)		8	7	(2)	38	55	**200**
	Rural	(1)	17		4		12	9	2	18	44	**697**
	All	3	20	(1)	3		11	9	2	23	47	**897**
												
Liberia 2007	Urban	11	29	15			24	(3)	(5)	39	77	**293**
	Rural	4	22	23			18	16	10	26	79	**721**
	All	6	24	20			20	12	9	30	78	**1,014**
												
Mali 2006	Urban	(3)	25	5			(2)	8	(2)	28	42	**325**
	Rural		14	(1)			3	8	4	14	28	**1,334**
	All	(1)	16	2			3	8	3	16	31	**1,660**
												
Niger 2006	Urban	(4)	22	(7)			(8)	(3)	(2)	26	43	**242**
	Rural		14	(1)			12	3	2	14	32	**1,576**
	All	(1)	15	2			11	3	2	16	33	**1,818**
												
Nigeria 2008	Urban	17	11	12	(1)		28		(1)	28	70	**608**
	Rural	8	13	6	2		31	2	2	22	63	**1,922**
	All	10	13	7	2		30	2	2	23	64	**2,530**
												
Senegal 2010–2011	Urban	10	21	4	(1)		7	3	5	31	48	**978**
	Rural	3	30	(1)	(1)		4	5	5	32	47	**1,268**
	All	6	26	2	(1)		5	4	5	32	48	**2,246**
												
Sierra Leone 2008	Urban	(5)	24	16	(3)		10	(1)	(2)	29	61	**153**
	Rural	5	35	5	3		4	4	(2)	40	54	**523**
	All	5	32	8	3		5	3	2	37	55	**676**
												
***Southern Africa***												
												
Lesotho 2009	Urban	24	(6)	(20)	(3)	(4)			(6)	29	59	**78**
	Rural	6	25	7	6	12	(1)	(4)	(2)	31	59	**297**
	All	10	21	9	5	10	(1)	(3)	(3)	31	59	**375**
												
Madagascar 2008–2009	Urban	(2)	26	24			(4)		(5)	28	57	**151**
	Rural		25	6			(2)	(2)	(2)	26	38	**841**
	All		25	9			(2)	(2)	3	26	41	**993**
												
Malawi 2010	Urban	25	25	(3)		4	5	(1)	(3)	50	63	**467**
	Rural	9	43	5		9	7	2	2	52	73	**2,691**
	All	12	40	4		8	7	2	2	51	71	**3,158**
												
Namibia 2006–2007	Urban	22	39	8			(3)	(1)		57	67	**243**
	Rural	17	39	(3)			(1)	(4)	(1)	54	63	**334**
	All	19	39	5			(2)	(3)	(1)	55	65	**577**
												
Swaziland 2006–2007	Urban	(11)	(24)	(11)	(2)	(22)	(9)	(2)		35	73	**52**
	Rural	(4)	50	5	(1)	16	(2)	(2)	(1)	53	76	**292**
	All	5	46	6	(1)	17	(3)	(2)	(1)	50	75	**343**
												
Zambia 2007	Urban	(7)	47	(3)			(3)			54	60	**291**
	Rural	(2)	51	(1)	(2)	5	3	7	(2)	52	68	**619**
	All	3	50	2	2	4	3	5	2	53	65	**911**
												
Zimbabwe 2010–2011	Urban	(1)	35	(2)	(1)		(3)			35	40	**230**
	Rural	(1)	29		(1)	(4)	(1)	(2)	(2)	30	39	**458**
	All	(1)	31	(1)	(1)	(3)	(1)	(1)	(2)	32	40	**688**
												
***East Africa***												
												
Ethiopia 2011	Urban	(2)	34	23		(1)	(5)			35	58	**158**
	Rural		22	6		(1)	2	1		23	33	**1,326**
	All		24	8		(1)	3	1		24	36	**1,483**
												
Kenya 2008–2009	Urban	18	15	13		(3)	12			32	59	**169**
	Rural	8	33	7	(1)	(2)	8	5	(1)	40	60	**740**
	All	10	29	8	(1)	2	9	4	(1)	39	60	**909**
												
Rwanda 2010	Urban	(1)	24	(5)	(6)		(8)	(4)	(3)	25	48	**140**
	Rural		24	(1)	14		4	3	8	24	50	**992**
	All		24	2	13		4	3	7	24	50	**1,132**
												
Tanzania 2010	Urban	10	30	(2)			14		11	39	63	**276**
	Rural	2	44				16		4	46	64	**833**
	All	4	41				15		6	44	64	**1,109**
												
Uganda 2011	Urban	(11)	(14)	47	(2)		(4)			25	74	**237**
	Rural	4	28	41	(1)		3	(1)	(1)	32	77	**1,528**
	All	5	26	42	(1)		3	(1)	(1)	31	77	**1,766**
												
**Survey – DHS Asia**												
												
Bangladesh 2011	Urban	(7)	(8)	45		(1)	(15)	(1)		(15)	75	**70**
	Rural	(2)	8	40			24	(4)		10	76	**318**
	All	(3)	8	41		(1)	22	(3)		11	76	**388**
												
Cambodia 2010	Urban	13	14	45		1	22		10	27	78	**131**
	Rural	5	24	40			16		20	28	74	**1,029**
	All	6	22	41			16		19^b^	28	75	**1,161**
												
India 2005–2006	Urban	13	4	61			3	2	(1)	16	80	**1,338**
	Rural	6	10	44	1		6	2	3	16	68	**4,465**
	All	7	9	48	1		5	2	3	16	71	**5,802**
												
Indonesia 2007	Urban	(1)	18	21	4		19		17	19	77	**799**
	Rural	2	19	8	12	1	17	2	17	20	72	**1,381**
	All	1	19	13	9	1	18	2	17^c^	20	74	**2,180**
												
Nepal 2011	Urban	(11)	(4)	29	(1)		(26)			(15)	69	**65**
	Rural	2	20	13	3		23		1	22	61	**646**
	All	3	18	15	3		23		1	21	62	**711**
												
Pakistan 2006–2007	Urban	7		64			(2)	7	(2)	8	78	**325**
	Rural	7	2	45	(1)		3	3	10	9	69	**732**
	All	7	2	51			3	4	8	8	72	**1,058**
												
Philippines 2008	Urban	5	21	12				7	(1)	25	45	**266**
	Rural	4	19	10			(1)	6	(2)	23	38	**294**
	All	4	20	11				6	2	24	41	**560**

a N values are adjusted (see Methods section). Note that point estimates for proportions based on adjusted Ns of less than 25 are indicated in parentheses, indicating a lower level of precision.

b This consisted mostly of “home of trained health worker.”

c This consisted mostly of community nurse-midwives.

Abbreviations: CHW, community health worker; HF, health facility; NGO, nongovernmental organization.

**Table 4.** Quality of ARI Care by “Received Antibiotics” (%)

**Survey**		**“Appropriate” provider**		**“Non-appropriate” provider**	
		**Sought care (A)**	**Received antibiotic**	**Sought care (B)**	**Received antibiotic**
			**(as a % of A)**		**(as a % of B)**
***Africa***					
Ethiopia	2011	27	70	2	(60)
Ghana	2008	51	35	14	(16)
Rwanda	2010	50	74	12	(28)
Senegal	2010–2011	50	50	10	(26)
Swaziland	2010	73	21	5	0
Uganda	2011	79	54	5	(49)
Zimbabwe	2010–2011	48	47	7	51
***Asia***					
Bangladesh	2011	35	83	47	79
Nepal	2011	48	37	29	61

Values in parentheses are based on cell sizes of less than 25.

**Table 5.** Quality of Diarrhea Care by “Appropriate” vs. “Non-Appropriate” Provider (%)

**Survey**		**“Appropriate” Provider**		**“Non-Appropriate” Provider**	
		**Pills/syrups for non-bloody diarrhea**	**ORS**	**Pills/syrups for non-bloody diarrhea**	**ORS**
***West and Central Africa***					
Benin	2006	-	63	-	14
DR Congo	2007	-	48	-	43
Ghana	2008	56	70	54	36
Guinea	2005	-	68	-	11
Liberia	2007	34	73	21	46
Mali	2006	-	50	-	4
Niger	2006	-	53	-	5
Nigeria	2008	62	51	57	20
Senegal	2010–2011	68	47	58	16
Sierra Leone	2008	75	84	78	59
***Southern Africa***					
Lesotho	2009	62	75	(44)	(10)
Madagascar	2008–2009	73	43	61	8
Malawi	2010	49	83	44	61
Namibia	2006–2007	36	87	(39)	(35)
Swaziland	2006–2007	61	94	(70)	58
Zimbabwe	2010–2011	49	45	(69)	(11)
Zambia	2007	50	86	67	26
***East Africa***					
Ethiopia	2011	50	62	52	(26)
Kenya	2008–2009	54	87	50	72
Rwanda	2010	62	64	57	(12)
Tanzania	2010	66	64	67	45
Uganda	2011	65	54	58	29
***Asia***					
Bangladesh	2011	-	87	-	77
Cambodia	2010	62	50	73	27
India	2005–2006	65	36	67	15
Indonesia	2007	74	56	80	26
Nepal	2011	48	63	66	42
Pakistan	2006–2007	65	56	61	37
Philippines	2008	45	76	(37)	(54)

Values in parentheses are based on cell sizes of less than 25.

“-” is indicated for surveys for which a general question on types of treatment received for diarrhea was not asked.

Abbreviations: ORS, oral rehydration solution.

### Acute Respiratory Infection

#### Care-Seeking Levels

For ARI, in 31 of the 42 countries for which data are presented here, care is sought from any source for at least 60% of reported cases. In 8 countries (Burundi, Central African Republic, Lao People's Democratic Republic, Madagascar, Mali, Mauritania, Sierra Leone, Zimbabwe), care seeking is somewhat lower, in the range of 40%–59%. Care seeking is much lower in 3 countries (Ethiopia, Somalia, and Togo), at around 30%. In most of the countries considered here, a relatively small proportion of care is sought from categories of source assumed to not be medically qualified (shops and non-allopathic providers). But in 9 of these countries, more than 30% of care is sought from such sources. Six of these countries are in western and west-central Africa (Benin, Côte d'Ivoire, the Democratic Republic of Congo, Mali, Niger, and Nigeria; the others were Bangladesh, Nepal, and Somalia).

#### Asia

The specific categories of provider from which care is obtained vary considerably by country, but certain patterns are evident. This is best appreciated by referring to the map (see Figure). In the Asian countries included here, care is obtained predominantly from the private sector, except in the 2 Central Asian Republics and Vietnam (where the public sector is the primary source); and in the Philippines, which has a mixed, public-private picture. In most of those countries where the private sector predominates, private clinicians are reported as the main source, although in Indonesia, drug shops were equally important, and in Nepal they were the most widely used source. The one Asian country included here in which community health workers (CHWs) are a significant source of care (for purposes of this paper, “significant” is used for cases in which 10% or more of cases sought care from a particular source) was Indonesia, where care was sought from village “health cadres” for 12% of cases.

#### Africa

In general, the public sector plays a more important role as a provider of ARI care in Africa than in Asia. But this is by no means the case in all countries. In 6 of the 16 western and west-central African countries included, more cases were treated outside than inside the public sector, with the private sector accounting for a particularly high proportion in the Democratic Republic of Congo, Niger, and Nigeria (the other countries in this region with predominantly private provision were Benin, Côte d'Ivoire, and Guinea). Among these countries, private-sector care consists mainly of care from shops, with the exception being the Democratic Republic of Congo, where private clinicians are also an important source. In several countries in this region, non-allopathic providers are an important source (Benin, Côte d'Ivoire, Guinea, and Mali). In several other countries where the public sector accounts for the largest proportion, private providers are also an important source, notably in Ghana and Liberia. In no countries in this region were CHWs a significant source of care (at least at the time of the most recent surveys), including Senegal, which was one of the first countries in Africa to scale up “community case management” (CCM). In southern and eastern Africa, in general, the public sector is the most important source for ARI care, but the picture varies across countries. In Kenya and Tanzania, although the public sector is the major source, drug shops are also important. Similarly, while the public sector is the major source in Madagascar and Namibia, private clinicians are also an important source. Uganda and Somalia are the 2 countries in this region considered in this analysis where the private sector is the main source of ARI care, with private clinicians providing care in Uganda, and shops filling that role in Somalia. Rwanda is the one country in this region where CHWs were a significant source of care at the time of the most recent survey (they were not in Madagascar or Malawi, where CCM has recently been widely implemented).

The public sector plays a more important role as a provider of ARI care in Africa than in Asia.

#### “Appropriate” vs. “Non-Appropriate” Care

For a small number of the surveys, we also had access to information on receipt of antibiotics, which is presented here disaggregated by “appropriate” vs. “non-appropriate” categories of provider (Table 4). Among those seeking care for ARI from medically qualified health workers, the proportion reporting receiving antibiotics varied considerably, with only 21% of such cases receiving antibiotics in Swaziland vs. 83% in Bangladesh. Among this small set of countries, only in Bangladesh and Nepal did “non-appropriate” providers constitute a significant source of care for ARI. Somewhat surprisingly, in Nepal, cases seen by such providers were far more likely to be treated with antibiotics than those seen by “appropriate” providers. In this instance, “non-appropriate” providers consisted primarily of drug shops and, as other investigators have documented,^5^ most cases treated in this sub-sector were in fact assessed at the “shop” by some category of health worker.

#### Urban-Rural Disparities

For care seeking for ARI, in most countries there were moderate urban-rural disparities (5%–10% lower level of care seeking from any source, in rural areas); however, in 5 of the countries included here, the disparity was greater than 20% (Ethiopia, Guinea-Bissau, Lao, Rwanda, and Somalia), and in 8 countries care seeking was approximately equally common in rural and urban areas (the Gambia, Ghana, Indonesia, Mozambique, the Philippines, Tajikistan, Togo, and Zambia). In most countries, private sources were more important in urban than in rural areas.

### Fever

#### Care-Seeking Levels

As a symptom of childhood illness, fever is of particular public health significance in countries with more heavily endemic malaria. Among the countries for which data were analyzed for this paper, care seeking for fever, from any source, was at similar levels as for ARI (Table 2). In 20 of the 29 countries, 60% or more of cases sought care. In 8 countries (India, Madagascar, Mali, the Philippines, Rwanda, Senegal, Sierra Leone, and Zimbabwe), 40%–59% sought care. In Ethiopia, a much lower proportion (26%) sought care—although malaria is not a major public health problem in most of Ethiopia. The relatively low level of care seeking is of particular concern in those countries where malaria makes up a significant proportion of childhood deaths, notably Mali, Senegal, and Sierra Leone. As with ARI, in most countries non-medically qualified providers accounted for a small proportion of care. In 3 countries, however, they made up 30%–50% (Benin, Guinea, and Nigeria), and in Bangladesh (where malaria is not a significant public health problem) such providers accounted for over 60% of care. Categories of provider consulted were similar to those seen for ARI.

Low levels of care seeking for fever is of particular concern in countries where malaria makes up a significant proportion of childhood deaths.

#### Asia

Among the Asian countries included, only in the Philippines was the public sector the principal source of care. Private clinicians were the main source of care in India and Pakistan. Bangladesh, Cambodia, and Nepal showed mixed pictures, with shops and private clinicians contributing. Non-allopathic providers were an important source in Bangladesh. Only in Indonesia were CHWs (“village health cadres”) a significant source.

#### Africa

In 6 of the 10 *western and west-central African countries*, the private sector was the main source of care for fever. In Liberia, clinicians were the main private source; in the Democratic Republic of Congo, shops and private clinicians were equally important. In the others (Benin, Guinea, Niger, and Nigeria), private shops/vendors predominated. Non-allopathic providers were a significant source in Benin, Guinea, and Mali. In the remaining countries in this region, the public sector was the main source, with contributions from the private sector. NGO/religious sources were an important contributor in Niger. CHWs were not a significant source in any of these countries.

In *southern and eastern* Africa, the picture was similar to that for ARI. The public sector accounted for the largest proportion of cases seen except in Uganda, where private clinicians predominated. Drug shops were an important source in Kenya and Tanzania, as were CHWs in Rwanda. Religious/NGO institutions were a significant source in Lesotho and Swaziland.

#### Urban-Rural Disparities

Urban-rural disparities for fever differed from those seen with ARI; many countries (12) had essentially equally high care-seeking rates in urban and rural areas. Only 1 (Sierra Leone) had a gap of more than 20 percentage points. As with ARI, the private sector was generally a more important source in urban settings than in rural ones.

### Diarrhea

#### Care-Seeking Levels

Although in most countries care for diarrhea (Table 3) was sought outside the home for most cases, such care seeking was less frequent than for ARI or fever. In 10 of 29 countries considered here, less than half of cases received care outside the home. This included several western and west-central African countries that were also noted to have lower levels of care seeking for ARI or fever, notably the Democratic Republic of Congo, Guinea, Mali, Niger, Nigeria, and Senegal. Other countries where less than half received such care were Ethiopia, Madagascar, the Philippines, and Zimbabwe. Non-medically qualified providers accounted for 30%–50% of cases in many of the western African countries (Benin, Ghana, Guinea, Liberia, Mali, Niger, and Nigeria) as well as in Bangladesh and Nepal.

Bangladesh had the highest proportion of cases treated with ORS. However, the other countries with high ORS use (Kenya, Malawi, Namibia, and Sierra Leone) relied relatively little on non-medically trained providers. Community health workers were a significant source of diarrhea care in the Democratic Republic of Congo and Rwanda. ORS use was especially low (<25%) in Madagascar, Mali, Senegal, and Zimbabwe where most diarrhea treatment is done in the public sector; in Niger, with mixed provision; and in Benin, where most such care is in the private sector.

#### “Appropriate” vs. “Non-Appropriate” Care

Two dimensions of appropriateness or quality of diarrhea care were investigated (Table 5):

- The dispensing of pills and syrups other than zinc for non-bloody diarrhea (most often antibiotics or anti-motility drugs), which is not in compliance with WHO guidelines
- The use of ORS for all diarrhea cases as a positive indicator of quality

In 18 of 22 countries, most episodes of non-bloody diarrhea cared for by “appropriate,” presumably medically qualified, providers received pills or syrups. Liberia and Namibia performed relatively better; only about one-third of such cases were given such non-recommended treatment.

On a more positive note, in the same number of countries (18 of 22) most “appropriate” providers dispensed ORS. A very high proportion dispensed ORS in Kenya, Namibia, Sierra Leone, and Zambia. In India, however, of cases seen by “appropriate” providers, only 36% received ORS (in Madagascar, Senegal, and Zimbabwe; also, most cases seen by “appropriate” providers did *not* receive ORS).

Among “non-appropriate” sources, rates of provision of pills and syrups were similar to those for medically qualified providers. However, “non-appropriate” providers generally offered ORS much less frequently than did the medically qualified ones. In several countries, notably in Kenya, the Philippines, and Uganda, reported ORS-use rates were higher than care-seeking rates, possibly reflecting household management with ORS supplies regularly kept on hand.

#### Urban-Rural Disparities

Urban-rural disparities in care seeking were smaller for diarrhea than for the other 2 conditions, with 14 countries having essentially equal rates, and 2 (Malawi and Zimbabwe) having higher care seeking in rural than urban areas. Only Ethiopia had an urban-rural gap of greater than 20 percentage points. As with the other conditions, the usual pattern was greater use of private sources in urban than in rural areas.

## DISCUSSION AND CONCLUSIONS

### Why This Analysis

Other investigators have done similar analyses in the past, looking at care seeking in individual countries^2^–^5^ and based on multi-country analysis of DHS data.^6^ In unpublished work, Montagu and Visconi^7^ have recently done such analysis looking specifically at care seeking from the public sector vs. private sector (further sub-divided as formal vs. informal), disaggregating by wealth quintile. Their analysis, while useful in exploring policy-relevant equity issues, provides less immediately useful information for program managers interested in developing a contextually strategic approach to improving care among the populations they are serving.

There are limitations to the analysis presented in this paper. As is evident in the tables, for some of the surveys the samples are quite small; therefore, the point estimates lack statistical precision (this has been flagged by giving estimates based on a cell size of less than 25 in parentheses). The oldest of the surveys included date to 2005 and 2006, so for some countries the data presented here may not adequately represent the current situation. It is likely that in some surveys there is substantial misclassification. For example, the provider in a peripheral dispensary or health hut may be a cadre of community health worker or health auxiliary but cannot be identified as such through the currently available data. Likewise, in some settings^5^ the shops that caregivers report getting treatment from are, in effect, outpatient clinics where children are typically examined and the treatment decision is made by the health worker at the “shop,” not by the household caregiver. Nevertheless, these disaggregated survey findings provide additional useful information to the program developer, over and above what is routinely presented in DHS and MICS survey reports. The 5 country boxes give examples of how these data can help guide program strategy and prioritization.

### Why Care-Seeking Patterns Are Important

Current patterns of care seeking need to be an important consideration in the development of effective approaches to improve case management for childhood illness at population scale. For example, in a given setting, if seeking care from any source is particularly uncommon in rural areas, strategies should be developed to address the actual barriers to such care seeking—for example, task shifting case management to auxiliary health workers to bring services closer to the population. If there is a high rate of care seeking from private practitioners, it may be appropriate to investigate quality of care, as has been done in some studies,^8^ and, if serious problems are found, to develop strategies targeting quality of care in the private sector. Likewise, if shops are a major source of care, social marketing or social-franchising approaches could be appropriate to help ensure appropriateness of care.

We are fortunate that DHS and MICS data sets already include useful information on this issue. Every time this information has been needed at country level, however, it has required further secondary analysis. In this paper, we have addressed this gap, making such information available for program managers across this set of 42 countries (indeed, this is the main objective of this study). We believe that similar disaggregated analysis should be routinely included in subsequent DHS and MICS reports.

Disaggregated analysis of care-seeking behavior for child illness should be routinely included in DHS and MICS reports.

### Need to Know More About Actual Care Provided

Although this analysis provides useful additional understanding of care-seeking patterns, clearly more is required to inform the development of strategies that are optimally responsive to the local context. Beyond knowing where caregivers are seeking treatment or advice, we need more information on the actual care provided. This paper has made available further analysis using DHS data from a limited number of countries, giving some indication about the quality of care provided, disaggregating by sources of care considered “appropriate” or “non-appropriate.” In certain settings, patients may be obtaining suitable care from “non-appropriate” or informal providers—for example, receiving ORS for diarrhea. Programmatic use of such a distribution channel may well be appropriate in some settings. However, this analysis has also demonstrated that even “appropriate” providers may be giving substandard treatment. For example, in almost all of the surveys it was found that most such providers dispense pills and syrups of various kinds (other than zinc) for non-bloody diarrhea. Whereas, in Bangladesh, fully 77% of those going to “non-appropriate” providers (shops and non-allopathic practitioners) received ORS, in India, only 36% of cases seen by “appropriate” or medically qualified providers received ORS. In Swaziland, relatively few cases of ARI seen by “appropriate” providers received antibiotics. This kind of country-specific information is needed by those tasked with determining the most effective strategies for improving sick-child care in their settings.

### The Role of Community Health Workers

Current global health program efforts in sick-child care focus mostly on “community case management,” that is, care provided by community health workers or health auxiliaries. In the analysis presented in this paper, care given by such providers represents a very small part of the service delivery mix. The Democratic Republic of Congo and Rwanda were the only countries of those analyzed where over 10% of cases of diarrhea were treated by CHWs. Only in Indonesia and Rwanda did CHWs treat more than 10% of ARI cases. In Nepal, where use of CHWs for such case management was pioneered and first taken to national scale, only 2%–3% of ARI and diarrhea cases are seen by CHWs, and most care is provided in the private sector. In Senegal, where CCM had expanded to cover almost all districts in the country by the time of the most recent DHS, CHWs were seen for only 1%–2% of cases of the 3 conditions. In Malawi, there has recently been significant CCM program expansion, but the last DHS predated this, so it does not reflect whatever relative contribution this program may currently be making. Nevertheless, the analysis presented here suggests that in most settings it may be inappropriate to focus program efforts on CHWs to the exclusion of other sources of care, which may, in fact, be much more widely used.

It may be inappropriate to focus program efforts on community health workers to the exclusion of more widely used sources of care.

### Context Matters

As with most public health problems, context matters in care seeking for childhood illness. Strategies that fail to take context into account are likely to be less effective. The first step in developing a strategy that is responsive to the actual situation on the ground is to understand what that situation is. In this case, that means understanding where caregivers are going when their children fall ill and what care is actually provided.

**BOX 1. Nepal (DHS 2011)**

Care seeking for ARI is high, particularly in Nepal's urban areas, with advice or treatment sought for close to 80% of cases (pneumonia is the leading cause of death among children under 5, beyond the newborn period, with diarrhea also an important cause but malaria not accounting for a significant proportion of deaths).^9^ Children are twice as likely to receive care in the private as in the public sector. This is the case in both urban and rural settings; however, whereas private health workers are more commonly cited as a source of care in urban areas, retail outlets and private health workers are used at similar levels in rural areas. Although not apparent from these data, other research^5^ has documented that in most instances when it is reported that care is sought from a shop, some assessment is made by a health worker at the shop before dispensing treatment, so they are in effect functioning as private clinics. Nepal has a well-established CCM program for childhood illness; however, the DHS data suggest that CHWs do *not* make up a significant fraction of the case-provision mix (and reported recourse to care from this source has declined since the 2006 DHS). Care seeking for fever shows a similar pattern to that for ARI, with a high level of care seeking, particularly in urban settings, and the private sector as the primary source. For diarrhea, care seeking is at a somewhat lower level overall and is lower in rural than in urban areas. As with ARI, the private sector is the most important source of care for diarrhea, particularly in urban areas.

Among those seeking care for diarrhea from “appropriate” or medically qualified providers, almost two-thirds reported receiving ORS versus a little under half among those seeking care from “non-appropriate” providers. “Appropriate” providers dispensed pills or syrups for half the cases of non-bloody diarrhea seen; two-thirds of cases seen by non-appropriate providers received pills or syrups. For ARI, those seeking care from “appropriate” providers were less likely to report receiving antibiotics than in most of the other countries for which we have data. However, most ARI care from “non-appropriate” providers included provision of antibiotics.

***Implications for Program Strategy:*** Although the public sector is a significant source of care, it plays a considerably smaller role than the private sector. Thus, to improve population health outcomes, efforts are warranted to identify and address gaps in the appropriateness and quality of care provided in the private sector. Antibiotic use by “appropriate providers” for ARI cases seen was relatively (perhaps inappropriately) low in comparison with that in other countries. Although the overall national picture shows relatively high levels of care seeking and a modest role for the public sector, there may be sub-populations or geographic areas showing quite different patterns. In these cases, a strategy focused on improving the coverage and quality of public sector provision (including use of community health workers) may still be appropriate.

**BOX 2. Ethiopia (DHS 2011)**

Ethiopia has very low levels of care seeking for all 3 conditions (the lowest of the 29 countries analyzed by DHS in this paper for ARI and fever, and among the lowest for diarrhea), particularly in rural areas. Despite very low care seeking, under-5 mortality is lower than in many of the other African countries in this analysis. Those children who do get care are likely, in the case of ARI, to receive antibiotics, and, in the case of diarrhea, to receive ORS (although other pills and syrups are also commonly given). Private-sector care in Ethiopia is provided by health professionals (not at shops). For ARI, care is twice as likely to be sought from the public sector than from the private. This is also true for fever and diarrhea, although the private sector plays a slightly more important role than for ARI. Pneumonia is the leading cause of death among children under 5 beyond the newborn period^10^ and therefore is an appropriate focus for program effort. Malaria does not account for a significant proportion of deaths.

***Implications for Program Strategy:*** With very low overall coverage and a minor role for the private sector, increasing coverage will require extending the public sector's reach more deeply at the community and household levels, particularly in rural areas, where most of the population lives. Although not reflected in the data presented here, this is the intent behind recent moves to increase sick-child care provided by health extension workers.

**BOX 3. Mali (DHS 2006)**

Mali continues to have very high under-5 mortality. At the time of the last DHS (2006), care seeking for ARI was at a moderate level and primarily from peripheral-level public-sector health facilities, although non-allopathic providers and drug shops were also significant sources of care. Care seeking for fever was comparatively low, with a wide gap between urban and rural coverage. This is notable, given high malaria mortality. Care for fever was sought predominantly from the public sector, although in urban areas some care was sought from retail outlets. Non-allopathic practitioners were a relatively important source of care for fever. Care seeking for diarrhea was lower than for any other country analyzed, with marked rural-urban disparity, and ORS use was very low. Non-allopathic practitioners were also consulted, although less frequently than for fever. Malaria, pneumonia, and diarrhea account for similar proportions of deaths among children under 5 beyond the newborn period,^11^ so all 3 warrant serious program attention.

***Implications for Program Strategy:*** Coverage was very low for diarrhea; rural areas, in particular, were not well reached. Since use of the private sector is low, it would be important to extend peripheral public health services more effectively at the community and household levels in order to achieve better population health outcomes (noting that, along with Somalia and Sierra Leone, Mali has the highest under-5 mortality^12^ of the 42 countries included in this analysis). Current efforts to expand access through a new cadre of community health workers appear to be an appropriate response in this situation. The relatively high use of traditional practitioners indicates that consideration could be given to working with this group to improve access to, for example, oral rehydration solution.

**BOX 4. Uganda (DHS 2011)**

Uganda is the one African country included in this analysis that showed a pattern of care seeking similar to that found in South Asia, relying primarily on private clinicians. For all 3 conditions, the proportion of cases of illness for which care was sought was high, and this was predominantly from “appropriate” providers, mainly private clinicians. Uganda was unusual among the countries included in this analysis in that in rural areas levels of care seeking were just as high as in urban areas, and, indeed, cases in rural areas were just as likely to be seen by private clinicians as in urban areas. Despite high levels of care seeking, child mortality is comparatively high. Quality of care appears to be an issue for diarrhea, as most cases of non-bloody diarrhea were treated with pills or syrups and fewer were given ORS. For ARI, slightly over half of those seen by “appropriate” providers reported having received antibiotics; this level may be compatible with appropriate use of antibiotics. Pneumonia is the number one cause of death among children under 5, beyond the newborn period, although malaria and diarrhea are also important causes.^13^

***Implications for Program Strategy:*** Based on this analysis, *access* to services is less of a problem in Uganda than in the other countries considered. Care is primarily provided in the private sector, by presumably medically qualified practitioners. However, this analysis suggests problems of quality of care, with too little use of ORS for diarrhea and inappropriate use of other remedies for non-bloody diarrhea. Accordingly, it would be appropriate for program efforts to be directed at improving quality of care given by private-sector providers of sick-child care, notably for diarrhea. All 3 of the major childhood conditions considered here are important causes of death and warrant serious program effort.

**BOX 5. Senegal (DHS 2010–11)**

Compared with rates in other countries in West Africa, under-5 mortality in Senegal is comparatively low.^14^ Malaria is the leading cause, followed by pneumonia and diarrhea.^15^ Care seeking from any source was lower than in most other countries considered here across all 3 categories of childhood illnesses. For ARI and fever, care seeking is somewhat lower in rural areas than in urban. Care-seekers relied primarily on public-sector providers, and non-medically qualified providers were not an important source. Senegal was one of the first countries in Africa to adopt and scale up management of childhood illness by community health workers (that is, CCM). By the time of the last DHS survey, this program had been implemented in 58 of the 69 districts in the country (personal communication with Serge Raharison). It is surprising, therefore, that for none of the 3 conditions are community health workers reported to have provided care for more than 2% of cases.

Oral rehydration salts were dispensed less frequently than in most other countries considered. Prescribing various pills and syrups was more common. Half of cases of ARI for which a medically qualified provider was consulted received antibiotics.

***Implications for Program Strategy:*** Since the private sector is not playing a prominent role in service provision, it would be sensible to focus program efforts on public-sector provision. The recent effort to expand access would seem appropriate given that care from any source has been lower than in many other countries (including some of Senegal's neighbors). However, the continued overall low level of care seeking and the very infrequent recourse to community health workers as a source of care suggests that there have been significant implementation problems with CCM. It would be warranted to investigate the factors that have contributed to the apparently poor performance of this program (such as availability of program commodities and acceptability of the providers) as well as the most significant barriers to obtaining care from public-sector health facilities. The relatively low rate of ORS dispensing also needs to be addressed.
